# Consolidation of Painted Plasters in Hypogean Environments: Comparative Performance of Inorganic Calcium-Based Products Under High-Humidity and Water-Saturated Conditions

**DOI:** 10.3390/nano16130831

**Published:** 2026-07-07

**Authors:** Roberta Cucchietti, Sara De Angelis, Eleonora Imperio, Vanessa Fontani, Lucia Conti, Giancarlo Sidoti, Sara Iafrate

**Affiliations:** 1 Wall Paintings Restoration Laboratory, Istituto Centrale per il Restauro (ICR), 00153 Rome, Italy; roberta.cucchietti@cultura.gov.it; 2 Department of Environmental Biology, Sapienza University of Rome, 00185 Rome, Italy; sara.deangelis-01@cultura.gov.it (S.D.A.); vanessa.fontani@cultura.gov.it (V.F.); 3 Materials Testing Laboratory, Istituto Centrale per il Restauro (ICR), 00153 Rome, Italygiancarlo.sidoti@cultura.gov.it (G.S.); 4 Non-Destructive Testing Laboratory, Istituto Centrale per il Restauro (ICR), 00153 Rome, Italy; eleonoraimperio@yahoo.it; 5 Physics Laboratory, Istituto Centrale per il Restauro (ICR), 00153 Rome, Italy

**Keywords:** calcium-based nanomaterials, nanolime, nanocalcite, hypogean environments, wall painting conservation, multi-analytical approach

## Abstract

Consolidation treatments are essential for the conservation of wall paintings affected by decohesion and disintegration phenomena. In hypogean environments, high relative humidity, limited ventilation and elevated biological risk impose particularly stringent performance requirements. Under these conditions, consolidants must ensure chemical compatibility, effective distribution within water-saturated substrates, long-term stability at high relative humidity and low toxicological impact. Calcium-based nanomaterials, especially nanolime dispersions, are widely employed as reference consolidants. However, their performance is strongly influenced by the dispersing medium, environmental conditions and substrate characteristics. This study addresses the lack of comparative assessments of currently available calcium-based consolidants by testing four products—two alcohol-based nanolimes (Nanorestore Plus^®^ and CaLoSil^®^), one aqueous nanolime dispersion (Nanolaq^®^) and a laboratory-formulated aqueous nanocalcite—applied to painted mock-ups. For the first time, the effectiveness of these treatments was investigated under both high relative humidity and water-saturated conditions through a multi-analytical approach. Colorimetric variations, water vapour permeability, water absorption and consolidant distribution within the pictorial layers were evaluated. The results provide a comparative assessment of consolidant performance as a function of the hygrometric regime of the substrate, with differentiated responses under high-humidity conditions and attenuated differences under water-saturated conditions. Overall, the effectiveness of the consolidant appeared to depend significantly on the combined influence of the dispersing medium, the imbibition state of the substrate and its chemical nature, highlighting the need for selection criteria and evaluation protocols based on simulating realistic conservation conditions.

## 1. Introduction

In conservation and restoration practice, consolidation refers to treatments intended to re-establish the material integrity of artefacts compromised by decohesion and disintegration phenomena [1], induced by physical, chemical or biological degradation processes [2,3]. The aim of the treatment is to improve the physico-mechanical properties of degraded substrates by restoring a more compact matrix without altering their original characteristics. According to conservation principles, an appropriate consolidation treatment must meet fundamental requirements, including compatibility with the constituent materials; effectiveness and adequate penetration capacity; moderate reduction in capillary water absorption; limitation of water uptake without impairing water vapour permeability; absence of chromatic or aesthetic alterations; and long-term stability and re-treatability to allow possible future interventions [4,5].

Within this general framework, hypogean environments represent one of the most challenging contexts for the consolidation of wall paintings. In such environments, the performance requirements for consolidants become more stringent and must be critically considered in relation to the severity of the conservation conditions, including high relative humidity (RH), water imbibition, poor ventilation and elevated biological risk. Under these conditions, consolidants must remain stable at high humidity levels and be capable of achieving effective distribution within water-saturated substrates. Indeed, the presence of water within the substrate may affect the distribution and arrangement of the consolidant within the pore network [6], making the choice of solvent a critical factor. Moreover, given the intrinsic vulnerability of these environments, an ideal consolidant should exhibit low bioreceptivity and, where possible, should be dispersed in solvents with low toxicological risk, particularly in poorly ventilated contexts [7].

As compatibility is considered a key criterion in consolidation treatments, inorganic consolidants represent the most appropriate choice for the conservation of lime-based wall paintings [5]. In this context, calcium-based systems are the most widely adopted, and nanolime dispersions based on calcium hydroxide currently represent the reference materials [8,9,10]. These systems can be broadly divided into two main categories: alcoholic formulations developed since the early 2000s [8,11,12] and, more recently, aqueous dispersions [13,14,15].

In addition to these materials, calcium carbonate nanoparticle suspensions (nanocalcite) have been investigated in academic research in both solvent-based [16,17] and aqueous formulations [18]. Although still scarcely available for wall painting conservation, recent commercial formulations based on CaCO_3_ nanoparticle dispersions have begun to emerge in other conservation sectors, such as paper deacidification [19]. Furthermore, the incorporation of CaCO_3_ particles into nanolime systems has also been proposed as a strategy to enhance consolidation effectiveness, mainly through pore-filling [20]. Nanocalcite is particularly promising due to its neutral pH, which does not induce the strong alkalinity typical of calcium hydroxide dispersions, thereby reducing the risk of chemical interaction with pigments sensitive to alkaline attack. Moreover, since the material is already in the carbonate form, it does not require carbonation times and may promote faster stabilization of the treated [21].

The widespread use of nanolimes is attributable both to their high chemical compatibility with lime-based materials [5,22,23] and to their ability to enhance the internal cohesion of the substrate without significantly affecting porosity or vapour permeability [24,25]. The effectiveness of nanolime treatments is strongly influenced by the dispersing medium, as well as by environmental and application parameters [9]. In particular, the type of solvent affects nanoparticle transport, deposition and carbonation processes [26,27], as well as the formation of different calcium carbonate polymorphs [28,29]. Several studies have investigated the relationship between penetration capacity and the type of dispersing medium, highlighting the tendency of nanoparticles to back-migrate toward the surface during solvent evaporation [30,31,32]. In addition to solvent-related factors, environmental and application parameters also play a significant role: high relative humidity (>75% RH), which promotes carbonation and whose kinetics may be accelerated by increased carbon dioxide availability [24,28,33]; the concentration of the dispersion applied [34,35]; the pore size distribution of the substrate [24,36]; and the application method and timing [37,38].

Since 2010, alcoholic nanolime dispersions have represented the most widely adopted approach for consolidation in hypogean environments [39,40,41,42]. However, the need for solutions specifically tailored to these contexts—where intervention sustainability and operator safety are critical—has highlighted several limitations related to the dispersing medium. In particular, the use of alcoholic solvents raises concerns regarding operator safety and biological risk [7,43], as short-chain alcohols may promote fungal spore germination under conditions of high humidity [44,45]. These issues have recently stimulated growing interest in aqueous nanolime formulations [46] and in alternative calcium-based systems such as nanocalcite, which may offer different reactivity and interaction with the substrate.

Despite the extensive literature on the development and application of nanolimes, only a limited number of comparative studies have directly addressed the differences between alcoholic and aqueous dispersions [47,48]. Virtually none have addressed these aspects under the controlled high-humidity or water-saturated conditions representative of hypogean environments, particularly with regard to the pictorial layer. Within this framework, the present study proposes a comparative evaluation of four calcium-based consolidants performed on paint layer specimens, with the aim of providing experimental data specifically tailored to the conservation of wall paintings in hypogean environments. The selected materials include the two most widely used alcoholic nanolimes (Nanorestore Plus^®^ and CaLoSil^®^), the recently developed aqueous dispersion Nanolaq^®^ and a laboratory-prepared aqueous nanocalcite dispersion.

Hypogean environments are frequently characterized by high-humidity conditions and partial pore saturation of the substrate. Since the effectiveness of consolidation treatments strongly depends on the hygrometric state of the substrate, the experimental design was based on the comparison of two different substrate conditions: non-saturated pores under high relative humidity (High-Humidity conditions, HH) and water-saturated pores (Water-Content conditions, WC). To investigate the influence of the chemical nature of the painted substrate on consolidant performance, two pigments were selected with different mineralogical composition. The performance of the products was compared in terms of colour changes, water transport properties, cohesion recovery and consolidant distribution within the pictorial layers.

## 2. Materials and Methods

### 2.1. Specimens

To reproduce pictorial layers representative of wall paintings, two series of low-cohesion painted plaster mock-ups were prepared using natural Siena earth (PS), a fine-grained pigment based on iron oxides and hydroxides, and artificial ultramarine blue (PB), a silica-based pigment with slightly coarser grains. The aim was to evaluate consolidant performance on heterogeneous pictorial matrices with different chemical and mineralogical compositions. XRD analysis performed on the pigment powders identified hauyne as the main constituent of the blue pigment, with trace amounts of chlorite, whereas Siena earth was found to be mainly composed of goethite. The corresponding XRD patterns are provided in Supplementary Figures S1 and S2. The average particle size was measured by optical microscopy, revealing mean grain sizes of approximately 2 μm for PS and 4 μm for PB.

The mock-ups consisted of a support mortar, formulated with finer aggregate fractions and a selected binder-to-aggregate ratio. It was prepared using a lime-based mortar, consisting of a 24-month-aged lime putty (CL90S, Bresciani Srl, Milan, Italy) and a Plio-Pleistocene calcarenite from the Montescaglioso area (Matera, Italy) as aggregate. The pigments (Martorelli Restauro & Colore Srl, Rome, Italy), dispersed in water, were applied by brushing 12 h after preparation of the plaster layer, when the mortar had already reached an advanced setting stage and carbonation had already significantly progressed.

For each specimen series, the complete mortar compositions, including aggregate granulometry, are reported in Table 1.

Representative images of the mock-up preparation procedure are provided in the Supplementary Materials (Figure S3).

Specimens measuring 13 × 13 × 1.5 cm were used for colorimetric measurements, water absorption tests by contact sponge and decohesion tests, whereas smaller specimens (5 × 5 × 1 cm) were used for water vapour permeability measurements. All specimens were cured for 90 days under controlled environmental conditions (T = 15 °C, RH = 88%).

### 2.2. Calcium-Based Products

Four calcium-based products were selected for the experimental study: three commercial nanolime formulations (Nanorestore Plus^®^, CaLoSiL^®^, Nanolaq^®^) and one laboratory-formulated dispersion (Nanocalcite).

Nanorestore Plus^®^ Propanol (CSGI, University of Florence, Florence, Italy) [48,49] and CaLoSiL^®^ IP (IBZ-Salzchemie GmbH & Co., KG, Halsbrücke, Germany) [48,50] are dispersions of calcium hydroxide nanoparticles in isopropanol. Nanolaq^®^ (SNAPTECH Srl, L’Aquila, Italy) [48,51] is an aqueous dispersion of calcium hydroxide nanoparticles reported by the manufacturer to contain a controlled residual chloride concentration of approximately 15 mg/L.

Nanocalcite is a laboratory-prepared aqueous suspension obtained by dispersing commercially available calcium carbonate nanoparticles (Skyspring Nanomaterials, Inc., Houston, TX, USA) in deionized water [52]. To enhance suspension stability, a quaternary ammonium salt Biotin T^®^ (Cts Srl, Altavilla Vicentina, Italy) [53] was added as a stabilizing agent (0.05 wt%).

The main characteristics of the four products are summarized in Table 2.

### 2.3. Testing Conditions

Two experimental moisture conditions were defined to simulate environments representative of hypogean settings and to evaluate the influence of substrate moisture content on consolidant performance: High Humidity (HH) and Water Content (WC).

Under HH conditions, the specimens were conditioned at 14–15 °C and 95–98% RH for 60 days prior to testing, promoting moisture uptake through water vapour penetration into the pore network.

Under WC conditions, the specimens were partially immersed in water for 1 h, then conditioned for 48 h at 14–15 °C and 95–98% RH and finally stored in individual sealed Polyvinyl Chloride (PVC) boxes (15 × 15 cm) to limit evaporation and maintain stable water content. Environmental parameters inside the boxes were maintained at approximately 15 °C and near-saturated (~100%) conditions.

**Figure 1:**
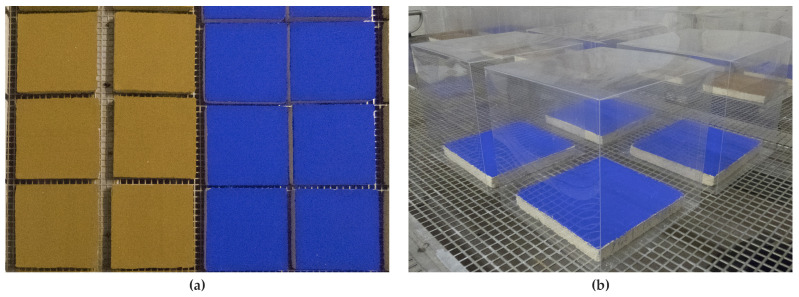
Representative specimens under the two experimental conditions: (**a**) HH conditions and (**b**) WC conditions.

Representative images of the specimens and the corresponding experimental moisture-conditioning setups are shown in Figure 1.

For each testing condition, specimen water content was determined by gravimetric measurements on three representative specimens. For HH conditions, specimen mass variation was measured before and after the 60 days of conditioning period. For WC conditions, mass variations were calculated between the pre-imbibition state and the end of the imbibition and subsequent conditioning procedure. Additional gravimetric measurement performed during the experiments confirmed that the specimen’s water content remained substantially stable over time under both conditions. Average values are reported in Table 3.

### 2.4. Application Protocol

The application protocol was defined to identify and standardize the most suitable procedure for consolidant application, based on conservation and aesthetic criteria related to surface modifications. The protocol consisted of two phases: preliminary tests to identify the most suitable surface pre-treatment and the subsequent consolidant application procedure.

#### 2.4.1. Preliminary Phase for Pre-Treatment Selection

Surface pre-treatments (pre-wetting) [39,54,55] have been reported in the literature as possible strategies for mitigating white haze formation, a phenomenon widely associated with nanolime-based treatments [56,57,58]. A preliminary testing phase was therefore conducted to compare different surface pre-treatment conditions. The methodology adopted for this assessment is described in Section 2.5.1, and the corresponding results are presented in Section 3.1.

Based on the results, IPA pre-treatment followed by complete solvent evaporation prior to consolidant application was selected under HH conditions, whereas no pre-treatment was adopted under WC conditions.

#### 2.4.2. Consolidant Application Procedure

The consolidant application procedure was defined according to the following parameters: consolidant concentration, total quantity of product applied, application technique and the number and interval of treatment cycles. All products were sonicated in an ultrasonic bath for 10 min prior to application. In accordance with the literature highlighting the effectiveness of low-concentration nanolime treatments [34,38], and supported by preliminary assessments, a protocol involving multiple applications at a concentration of 5 g/L was adopted. Low-pressure spraying was employed during the initial application cycles to minimize mechanical stress on the surface, whereas brush application was introduced only after sufficient recovery of surface cohesion, with Japanese tissue paper (12.3 g/m^2^) interposed as a protective layer. Application cycles were spaced 48 h apart under HH conditions and 72 h apart under WC conditions. Under HH conditions, an additional post-treatment by water spraying was carried out using low pressure.

To ensure uniform experimental conditions, all parameters were kept constant within each specimen series, except for the application method, which was adapted to the different moisture conditions (HH and WC).

To evaluate the influence of the dispersing media alone on surface modifications, control specimens were treated using only water, isopropylic alcohol (IPA) and water containing 0.05 wt% Biotin T, following the same procedures adopted for the consolidants. The overall application protocol adopted for each experimental condition, including both the selected surface pre-treatment and the consolidant application procedure, is summarized in Table 4 and Table 5.

### 2.5. Evaluation Methods

#### 2.5.1. Preliminary Test for Pre-Treatment Selection

To identify the most suitable surface pre-treatment for reducing white haze formation after consolidation, four pre-treatment conditions were compared: no pre-treatment, water pre-treatment, IPA pre-treatment, and acetone pre-treatment. The assessment was performed under both moisture conditions (HH and WC) using PB specimens, as the high contrast between the ultramarine blue paint layer and the white haze facilitated the detection and evaluation of whitening phenomena. After each pre-treatment, the four consolidants were applied at a concentration of 5 g/L in three treatment cycles.

As the objective of this assessment was protocol selection rather than a statistical evaluation of treatment performance, image analysis was conducted on a single representative sample for each condition. Results are therefore reported as single values and were used exclusively for comparative purposes.

The effects of the different pre-treatment conditions were evaluated through image analysis. Images acquired during the application trials were analyzed using ImageJ software (Fiji distribution, version 1.54g, National Institutes of Health, Bethesda, MD, USA) [59] through a pixel-based approach [60,61]. All images were acquired under identical lighting conditions at the same spatial resolution, then converted to 8-bit grayscale. White haze areas were isolated by applying the same grayscale threshold value (150–255) to all images to ensure consistency and reproducibility of the analysis.

The extent of whitening was quantified as percentage area (Area%), corresponding to the percentage of threshold-selected pixels in the analyzed image.

#### 2.5.2. Test for the Evaluation of Consolidation Treatments

For each consolidation treatment and experimental condition, the full set of tests was conducted on three independent specimens.

All tests were performed 30 days after treatment application, following indirect verification of a sufficient degree of carbonation through surface pH measurements, carried out using a portable pH metre equipped with a flat surface electrode (XS Instruments). After 30 days, pH values had returned to levels comparable to those recorded before treatment, indicating that carbonation had progressed to a sufficient extent for the subsequent testing phase (Supplementary Tables S1 and S2).

Due to the intrinsic variability associated with both the manual preparation of the mock-ups and the induced decohesion procedure adopted during specimen preparation, normalized parameters expressed as relative variations with respect to the initial untreated condition of each specimen group (UT) were adopted whenever applicable. This approach was intended to minimize specimen-to-specimen heterogeneity and allow a more reliable comparative evaluation of treatment performance under the different experimental conditions.

Considering the intrinsic heterogeneity of the experimental specimens, the comparative evaluation of treatment performance primarily focuses on mean values and overall trends observed among the different formulations.

The performance of the consolidation treatments was evaluated using the following methods:

- Colourimetric measurements.

Colourimetric measurements were performed to evaluate surface colour changes induced by the treatments. Colour variations were evaluated through the total colour difference ΔE*Lab, while the hue difference ΔH*ab was additionally considered to reduce the influence of moisture-related lightness variations during measurements. Measurements were carried out by spectrophotometry in the CIE L*a*b* and CIE L*C*h* colour space, in accordance with CIE 1976 [62] and UNI EN 15886:2010 [63], using CM-700d spectrophotocolorimeter (Konica Minolta Inc., Tokyo, Japan) with standard illuminant D65, a 10° observer and an 8 mm measuring aperture (MAV). For each specimen, three repeated measurements were performed on the same measurement spot, and the final colour values were calculated as the average of the three measurements.

- Water absorption (Contact sponge method).

The influence of the consolidation treatments on water absorption was evaluated to detect possible variations in surface wettability and capillary behaviour. Measurements were carried out using the contact sponge method, in accordance with UNI 11432:2011 [64], employing the C2 device (wet sponge diameter: 2.7 cm). The sponge was pre-wetted with 1 mL of deionized water prior to each measurement and kept in contact with the surface for a fixed time of 5 min. Water absorption was determined by gravimetric analysis using an analytical balance (resolution: 0.1 mg). Measurements were performed on three different areas of each specimen. Prior to testing, specimens were not conditioned at 60 °C as prescribed by the standard, in order to preserve the moisture conditions established during the experimental treatments.

Water absorption results are expressed as relative variation (ΔWa, %) with respect to the initial untreated condition of each specimen, calculated according to the following formula:

(1) $$\Delta Wa\left(\%\right)=100\times \frac{\left({Wa}_{treated}-{Wa}_{untreated}\right)}{{Wa}_{untreated}},$$

- Surface cohesion evaluation.

Due to surface moisture and capillary saturation of the paint layers under the adopted testing conditions, performing the Scotch Tape Test (STT) [65,66] to assess treatment effectiveness was not feasible. Adhesion of the Scotch tape to the surface was inhibited by the moisture and water content of the specimens. To overcome this issue, material loss variation before and after treatment was evaluated by a controlled abrasion test, consistent with surface chalking assessment criteria defined in ASTM D4214 [65,67,68]. The test (hereafter referred to as the Rubbing Test, RT) was performed using a custom compression-spring mechanical device (designed and built by Istituto Centrale per il Restauro, Rome, Italy) [46] designed to apply controlled pressure and rubbing action to the testing surface. The device consists of a hollow cylindrical body housing an internal sliding piston terminated by a circular end-face (3 cm in diameter). The piston is mounted coaxially with a steel compression spring, which generates a constant and reproducible load when the device is pressed against the surface. The circular end-face acts as a support for interchangeable test pads (2.5 cm in diameter), composed of a 5 mm thick polyurethane (PU) foam layer coupled with lightweight tissue paper (12.5 g/m^2^). This multilayer pad system ensures homogeneous contact distribution between the device and the testing surface while accommodating minor surface irregularities. During operation, the spring applies a preset pressure of 126 g/cm^2^ to the surface. The rubbing action is generated by compressing the spring and simultaneously rotating the piston by 180° around its longitudinal axis, thus ensuring controlled abrasion conditions throughout the test.

The amount of material removed during the test was determined by gravimetric analysis of the multilayer pad using an analytical balance (resolution: 0.1 mg). The removed mass was used to calculate the decohesion index (DI) [69,70], defined as the ratio of the removed mass to the contact area of the test assembly. Results are expressed as relative variation (ΔDI, %) with respect to the initial untreated condition of each specimen, calculated as:

(2) $$\Delta DI\left(\%\right)=100\times \frac{\left({DI}_{treated}-{DI}_{untreated}\right)}{{DI}_{untreated}},$$

- Water vapour permeability (wet-cup method).

The influence of the consolidation treatments on water vapour permeability was evaluated to assess their physical compatibility with the original substrate in terms of vapour-phase moisture transport. Measurements were performed according to UNI EN 15803:2010 [71] using the wet-cup method. A relative humidity gradient was established across the specimen by placing a saturated potassium nitrate (KNO_3_) solution inside the measuring cell, maintaining an internal relative humidity of approximately 93%, with the specimens placed in a climatic chamber set between 60 and 65% RH at 23 ± 0.5 °C. Despite instrumental limitations, this configuration still ensured a sufficient relative humidity gradient between the interior of the cups and the external chamber environment. Water vapour transport through the specimen was determined by gravimetric monitoring of the mass variation in the cup at 24 h intervals, using an analytical balance (resolution: 0.1 mg), under steady-state conditions. The vapour diffusion resistance factor (μ) is expressed as relative variation (Δμ, %) with respect to the initial untreated condition of each specimen, calculated as:

(3) $$\Delta \mu \left(\%\right)=100\times \frac{\left(\mu_{treated}-\mu_{untreated}\right)}{\mu_{untreated}},$$

Water vapour permeability measurements were carried out only under HH conditions, as the testing protocol requires controlled and stable moisture gradients. Such conditions cannot be reliably achieved in water-saturated substrates (WC), where the presence of liquid water interferes with vapour-phase transport measurements.

- SEM–EDS analysis.

Scanning Electron Microscopy (SEM, Zeiss EVO 60, Carl Zeiss AG, Oberkochen, Germany) coupled with Energy Dispersive X-ray Spectroscopy (EDS, Oxford Inca Pentaflex microprobe, AZtec software version 3.3 SP1) was employed to investigate the apparent penetration and distribution of the consolidants within the pictorial layers.

Polished cross-sections were obtained by embedding fragments of selected specimens (PB series) in polyester resin. SEM observations were performed operating in backscattered electron (BSE) mode at an accelerating voltage of 20 kV, in extended pressure at 100 Pa, without conductive coating.

Elemental analyses were performed by EDS through spot analyses, line scan profiles and elemental mapping. Line scans were acquired along directions perpendicular to the sample surface to assess the distribution of calcium (Ca) in relation to silicon (Si), the latter associated with the ultramarine blue pigment. The PB specimens were selected due to the presence of a silicon-rich pigment, which enables a clearer distinction between the pictorial layer and both the underlying substrate and calcium-based consolidant.

Line scan profiles of Ca and Si were normalized and used for qualitative comparison of elemental distribution across the cross-sections. This approach enabled visualization of the relationship between consolidant presence (Ca) and pigment distribution (Si) within the pictorial layer.

Ca elemental maps were additionally acquired to provide a spatial visualization of consolidant distribution within the cross-sections.

Semi-quantitative EDS analyses were performed on multiple selected areas of the cross-sections. For each specimen, three representative regions were identified: (i) the embedding resin, used as a background reference (Ca_resin_); (ii) the plaster substrate, considered as the reference for maximum calcium content (Ca_plaster_); and (iii) the pictorial layer, used to evaluate the relative degree of Ca enrichment associated with consolidant distribution (Ca_sample_).

The normalized Ca content of the pictorial layer was calculated as:

(4) $${Ca}_{norm\left(\%\right)}=\frac{{Ca}_{sample}-{Ca}_{resin}}{{Ca}_{plaster}-{Ca}_{resin}}\times 100,$$

This approach compensates for background signal contributions and instrumental variability, providing a relative estimation of calcium enrichment within the pictorial layer associated with consolidant distribution.

- Comparative performance ranking.

To enable a comprehensive comparison of consolidant performance under the two experimental moisture conditions, a qualitative ranking system was established based on the main parameters examined in this study to support the discussion of the results. Because the investigated parameters are expressed in different physical units, ranking intervals were determined separately for each parameter while following a common mathematical approach. For each test, the highest recorded value was identified and divided by six, corresponding to the six ranking levels adopted. The resulting intervals defined the lower and upper thresholds for each ranking category, providing a standardized basis for comparing the performance of the different consolidants (Supplementary Table S3). For values falling close to a threshold, assignment to the adjacent upper or lower interval was made to preserve overall comparability and ensure that the ranking accurately reflected the general performance trends observed among the consolidants.

## 3. Results

### 3.1. Evaluation of Pre-Treatment

**Figure 2:**
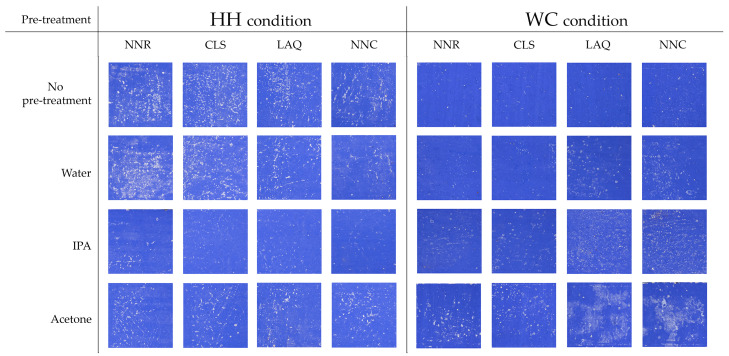
Effect of different pre-treatment conditions on white haze formation after consolidation treatment under both conditions.

Surface whitening under HH and WC conditions was evaluated comparatively through image analysis (Table 6 and Figure 2). Under HH conditions, pre-treatment with IPA reduced the extent of whitening compared to specimens without pre-treatment. Conversely, water pre-wetting and acetone pre-treatments showed more variable responses, with no consistent trend observed across the different consolidants.

Under WC conditions, specimens without pre-treatment exhibited the lowest, or among the lowest, whitening values for all consolidants. Pre-treatments generally increased the extent of white haze, with particularly pronounced effects observed for LAQ- and NNC-treated specimens following IPA or acetone pre-treatment.

### 3.2. Evaluation of Consolidant Treatments

#### 3.2.1. Colorimetric Changes

Colorimetric changes were evaluated in terms of ΔE* and ΔH* reported in Table 7 and Table 8. Detailed CIELAB parameters (ΔL*, Δa*, Δb*, ΔC*) are provided in Supplementary Tables S4 and S5.

Under HH conditions, PS specimens exhibited a differentiated behaviour depending on the dispersing medium. Alcohol-based formulations (NNR and CLS) maintained ΔE* values below perceptibility (ΔE* **<** 3). By contrast, aqueous dispersions (LAQ and NNC) produced markedly higher variations (ΔE* = 6.21 and 10.89), associated with increased lightness and reduction in the yellow component (Δb* < 0). In these cases, low ΔH* values indicate that colour changes were mainly driven by variations in lightness and chroma rather than hue rotation. A different behaviour was observed for PB specimens, which were markedly more sensitive to treatment. All consolidants induced substantial colour changes (ΔE* > 7), particularly the aqueous formulations LAQ and NNC (ΔE* = 12.42 and 16.56). These changes involved increased lightness (L*) and concurrent shifts in a* and b*, indicating hue rotation and reduced colour intensity.

Dispersing media alone did not produce significant colour variations in either pictorial system under HH conditions.

Under WC conditions, PS specimens remained chromatically stable for all treatments and dispersing media (ΔE* ranging from 0.53 to 1.47), with negligible hue variation. Conversely, PB specimens showed elevated ΔE* values for all treatments (ΔE* ranging from 6.47 to 12.18). In this case, variations were characterized by decreased lightness (ΔL* < 0) and increased a* and b* values, consistent with a shift toward purplish tones and reduced chroma. However, ΔH* values were higher in specimens treated with dispersing media alone (ΔH* = 7.14–7.91) than in consolidant-treated specimens (ΔH* = 2.52–3.80). In PB specimens, dispersing media alone induced significant colour variation (ΔE* ranging from 8.86 to 10.19).

#### 3.2.2. Water Transport Properties

Under HH conditions, all consolidants reduced water absorption across the substrates relative to UT specimens (Table 9). For PS specimens, reductions showed similar values for NNR, CLS and LAQ (56–61%), while NNC showed a more limited reduction (29%). For PB specimens, reductions were lower than those observed for PS, while maintaining a similar trend among the formulations, with the lowest reduction observed for NNC (12%). Treatment with dispersing media alone resulted in only minor variations across all substrates (ΔWa ≤ 22%).

Under WC conditions, all consolidants reduced water absorption in both painted specimens (Table 9). For PS specimens, ΔWa ranged from 46% to 60%, with the highest reductions achieved by aqueous formulations LAQ and NNC (59% and 60% respectively), whereas alcohol-based systems showed slightly lower values (NNR 46% and CLS 48%). For PB specimens, reductions were generally higher than those observed under HH conditions, with ΔWa ranging from 54% to 70%. Aqueous formulations showed the greatest reductions once more (LAQ 70%; NNC 63%), followed by alcohol-based products (NNR 54% and CLS 56%). Treatments with dispersing media alone resulted in relatively minor variations (ΔWa ≤ 24%).

In terms of vapour transport (Table 10), the vapour diffusion resistance factor (μ) decreased for all consolidants under HH conditions, ranging from −21% to −41% for PS and from −21% to −63% for PB. The most pronounced reductions were observed for PB specimens treated with alcohol-based formulations (NNR −63% and CLS −56%), whereas PS specimens showed more evenly distributed variations among the different formulations. Despite these variations, permeability value behaviour after treatment remained compatible with that typically observed in lime-based systems in all specimens.

#### 3.2.3. Surface Cohesion (Decohesion Index, DI)

Under HH conditions, all consolidants reduced the decohesion index (DI) compared to untreated specimens (Table 11). Alcohol-based systems generally exhibited the highest reductions, particularly for PB specimens (NNR 90% and CLS 91%), whereas aqueous dispersions showed slightly lower, yet still significant, reductions for both pictorial systems.

Under WC conditions (Table 11), the consolidating effect remained evident, although with generally lower values and less pronounced differences among products, respect to HH conditions. ΔDI ranged from 51% to 63% for PS specimens and from 48% to 72% for PB specimens, with comparable effectiveness observed between alcohol-based and aqueous systems. In particular, LAQ exhibited the highest reductions under WC conditions for both PS and PB specimens. For treatments with dispersing media alone, ΔDI variations under HH conditions were lower than those observed for the consolidants, indicating the absence of a significant contribution to the consolidating effect. Nevertheless, systematically higher values were observed in PB specimens compared to PS specimens. In particular, aqueous dispersing media exhibited the highest increases, whereas IPA showed the most limited effects in both specimen series. Under WC conditions, ΔDI values were overall lower than those observed under HH conditions, and aqueous dispersing media exhibited negative values.

#### 3.2.4. SEM–EDS Analysis

**Figure 3:**
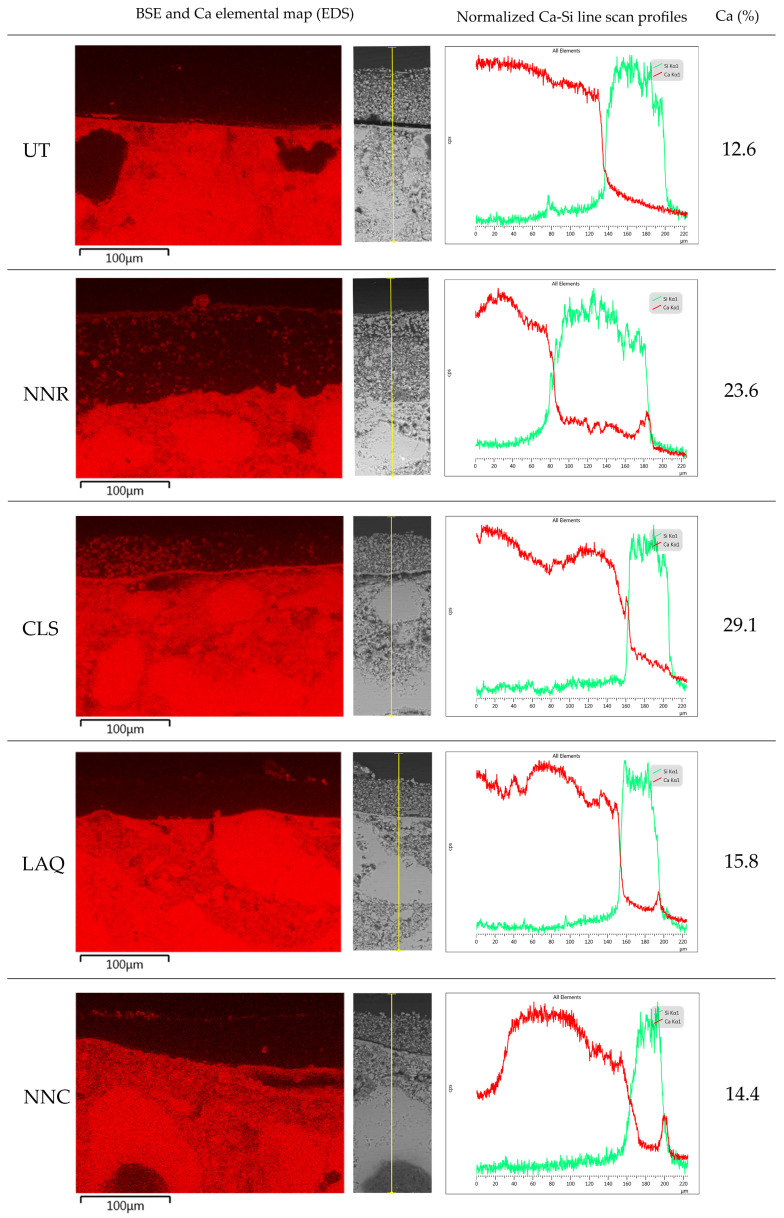
SEM–EDS analysis of consolidant penetration in PB specimens under HH conditions. Combined BSE and Ca elemental map images, normalized Ca and Si linescan profiles, and normalized Ca content (%). Red: Ca; green: Si. The yellow line indicates the scan path used for the normalized Ca–Si line profile.

**Figure 4:**
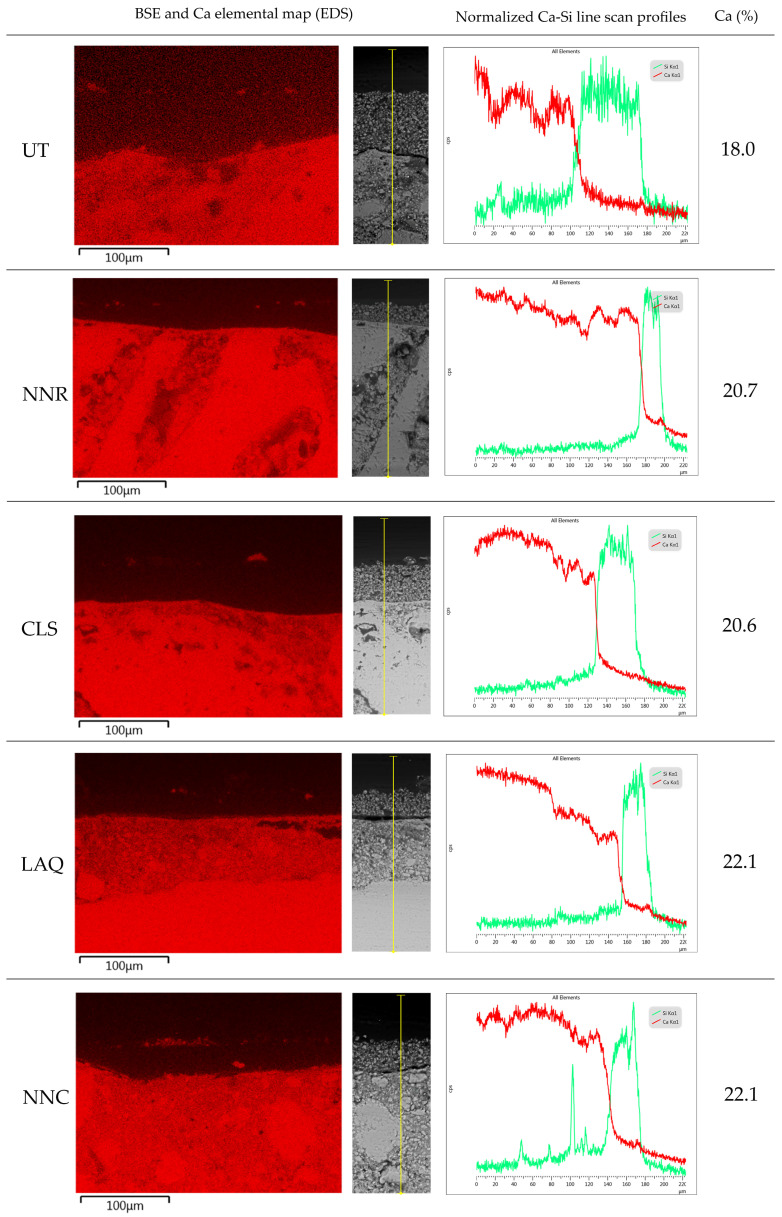
SEM–EDS analysis of consolidant penetration in PB specimens under WC conditions. Ca elemental maps, normalized Ca and Si line scan profiles, and normalized Ca content (%). Red: Ca; green: Si. The yellow line indicates the scan path used for the normalized Ca–Si line profile.

SEM–EDS analyses were carried out to evaluate the consolidant distribution and Ca enrichment within the pictorial layer of PB specimens under both HH and WC conditions (Figure 3 and Figure 4). The assessment was based on the combined interpretation of Ca elemental maps, normalized Ca and Si line scan profiles and normalized Ca content values.

Under HH conditions, clear differences in consolidant distribution were observed among the tested formulations. The UT specimen showed a baseline normalized Ca content of 12.6% together with a relatively uniform Ca distribution. Alcohol-based consolidants resulted in the highest Ca enrichment within the pictorial layer. CLS showed the highest normalized Ca content (29.1%), followed by NNR (23.6%). In both cases, line scan profiles revealed a marked increase in Ca signal within the Si-rich region compared to UT specimens, suggesting a more extensive distribution of the consolidant within the paint layer. However, differences in spatial arrangement emerged from the elemental maps. NNR displayed a more homogeneous distribution, with a more continuous arrangement along the observed pore network, whereas CLS exhibited a more heterogeneous and locally aggregated Ca distribution, with distinct accumulations in specific areas of the cross-section. Conversely, aqueous formulations produced lower Ca enrichment within the paint layer. LAQ showed a moderate increase in normalized Ca content (15.8%), with line scan profiles indicating a more limited Ca signal within the pictorial layer. NNC showed values (14.4%) comparable to the UT specimen, suggesting limited apparent distribution within the pictorial layer. Ca elemental maps indicated a more surface-localized distribution for both aqueous treatments, consistent with line scan profiles revealing Ca enrichment mainly confined to the near-surface region.

Under WC conditions, the UT specimen showed a higher baseline normalized Ca content (18.0%), compared to HH conditions, likely associated with differences in specimen preparation and conditioning procedures. All consolidants exhibited similar normalized Ca values, ranging between 20.6% and 22.1%. In contrast to HH conditions, aqueous formulations (LAQ and NNC) showed Ca contents comparable to, or slightly higher than, those of alcohol-based products (NNR and CLS). Line scan profiles revealed a more uniform Ca distribution across the cross-sections, with only slight differences in Ca signal within the Si-rich region among the different treatments. In several cases, a small increase in Ca signal was observed in the near-surface region. Although this feature was not clearly detectable in the elemental maps, line scan profiles suggest a Ca distribution more confined to the outermost regions of the pictorial layer.

Overall, SEM–EDS results highlight a strong influence of substrate moisture conditions on consolidant distribution within the paint layer. Alcohol-based systems showed greater apparent penetration under HH conditions, whereas differences among products were significantly reduced under WC conditions.

## 4. Discussion

### 4.1. Evaluation of Pre-Treatment

Pre-treatment modifies the imbibition state of the porous system, thereby influencing the distribution of the consolidant within the capillary network. Image analysis results indicate that the effectiveness of pre-treatment in mitigating white haze formation depends primarily on the degree of substrate saturation at the time of application and on the nature of the consolidant dispersing medium.

Under HH conditions, IPA pre-treatment resulted in comparatively lower whitening values for all investigated consolidants. This behaviour may be associated with the lower surface tension of IPA, which can influence substrate wettability and the distribution of the liquid phase during the early stages of consolidant application. However, whitening values observed during the final treatment procedures were generally higher than those recorded during the preliminary pre-treatment assessment. This difference may be related to the greater amounts of consolidant applied during the final testing protocol, which involved multiple application cycles. Under the same conditions, water pre-wetting increased surface whitening in alcohol-based systems (NNR and CLS), whereas comparatively lower whitening levels were observed for the aqueous dispersions (LAQ and NNC), suggesting that the response of the system varies according to the nature of the dispersing medium. In particular, water introduced into the substrate pore network may compromise the stability of the alcohol-based dispersion.

Under WC conditions, corresponding to a state close to capillary saturation, the introduction of additional fluids generally resulted in increased surface whitening, whereas the absence of pre-treatment considerably limited white haze formation. The more pronounced whitening observed for the aqueous dispersions after IPA or acetone pre-treatment further suggests that the introduction of organic solvents into a highly water-saturated system may temporarily influence the stability and distribution of the aqueous consolidant dispersion within the porous network, potentially favouring localized surface accumulation phenomena.

Overall, the results suggest that the effectiveness of pre-treatment is strongly dependent on the imbibition conditions of the substrate, which can either enhance or attenuate its influence on consolidant distribution and white haze formation.

### 4.2. Evaluation of Consolidant Treatment

To facilitate the comparative evaluation of consolidant performance under different moisture conditions, a ranking system (Table S3) was adopted in which the performance of each product was ranked based on the results obtained for the investigated parameters (Table 12 and Table 13).

The integrated analysis of the results highlights how the effectiveness of consolidating treatments is governed not only by the chemical and physical nature of both the consolidants and the substrate, but also by the interaction between the dispersing medium, the characteristics of the pictorial matrix, and the hygrometric state of the substrate [72]. Overall, differences among the formulations were more pronounced under HH conditions, whereas a partial convergence in performance was observed under WC conditions.

Under HH conditions (Table 12), alcohol-based formulations (NNR and CLS) showed the highest performance in terms of cohesion recovery, with ΔDI values consistently exceeding those measured for aqueous dispersions in both painted specimens.

The systematically higher ΔDI values observed for PB specimens also suggest a different response of the two pictorial matrices. In PS specimens, the colloidal behaviour of Siena earth may have promoted a more compact particle arrangement already during hygrometric conditioning. This may have contributed to a partial reduction in surface decohesion prior to treatment. Consequently, untreated PS specimens exhibited lower initial decohesion values than PB specimens, thereby limiting the measurable cohesion recovery after consolidation. This interpretation is also consistent with the results obtained for treatments with dispersing media alone, which systematically showed higher ΔDI values in PB than in PS specimens. SEM–EDS observations performed on PB specimens provide an additional interpretative element, revealing differences in Ca distribution consistent with the observed cohesion recovery behaviour. The greater Ca enrichment detected within the pictorial layer for alcohol-based systems suggests a more extensive distribution throughout the porous network. This performance was likely favoured by the lower surface tension of the alcoholic dispersing medium. This observation further supports the influence of the dispersing medium on nanoparticle transport and deposition within porous substrates previously highlighted in the literature [30,73]. At the same time, some differences in Ca distribution were observed between the two alcohol-based formulations. NNR exhibited a more homogeneous distribution, characterized by a continuous arrangement along the observed pore network, whereas CLS showed localized accumulations, suggesting a less homogeneous consolidant arrangement during deposition. Furthermore, such localized and superficial Ca accumulations could be related to surface accumulation phenomena associated in the literature with nanolime back-migration during solvent evaporation in alcohol-based dispersions [30,32]. By contrast, aqueous formulations (LAQ and NNC) displayed lower Ca enrichment within the pictorial layer coupled with a more surface-localized distribution. This indicates a less extensive distribution throughout the porous network compared to the alcohol-based systems. Such behaviour may also be associated with the lower colloidal stability of the laboratory-prepared nanocalcite dispersion (NNC), as well as with that previously reported in the literature for LAQ aqueous nanolime dispersions. Reduced colloidal stability may favour localized deposition and surface accumulation phenomena, thereby limiting penetration effectiveness [48].

From a colorimetric perspective, PB specimens exhibited the most pronounced chromatic variations for all treatments, particularly for aqueous dispersions. These variations, mainly associated with increased diffuse reflectance and reduced colour intensity, appear consistent with the more surface-localized Ca distribution observed by SEM–EDS. Conversely, PS specimens remained relatively stable after treatment with alcohol-based formulations, whereas aqueous dispersions produced variations above the perceptibility threshold, mainly associated with changes in lightness and saturation. The generally low ΔH values indicate substantial hue stability. By contrast, treatments with the dispersing media alone did not induce significant chromatic variations, supporting the hypothesis that the optical alterations observed after consolidation are mainly related to consolidant deposition and distribution within the pictorial layer.

Regarding water absorption, all formulations reduced water uptake, with relatively comparable behaviour observed in PS specimens, whereas greater variability emerged in PB specimens. In particular, NNC exhibited the least pronounced reductions in ΔWa in both pictorial matrices, suggesting a more limited effect on water transport properties compared to nanolime dispersions. This result may be associated with the different nature of the consolidant, which already consists of calcium carbonate and therefore appears less dependent on subsequent carbonation processes, with consolidation effectiveness being more strongly related to physical deposition phenomena within the porous network [48,74].

Water vapour permeability, evaluated exclusively under HH conditions, showed an increase in vapour diffusion resistance for all treatments. Although the absolute values remained compatible with those typically observed in lime-based pictorial systems, the observed variations indicate a modification of the porous system consistent with consolidant ingress and deposition within the pictorial matrix. Overall, these differences suggest that improved cohesion recovery does not necessarily correspond to a proportional modification of water transport properties.

Under WC conditions (Table 13), the high water content within the pore network appears to modify consolidant transport dynamics, in agreement with the role attributed in the literature to moisture and pore connectivity in nanolime transport processes [24]. The system response appears to be primarily governed by the hygrometric state of the substrate and by liquid-phase dynamics within the porous network. As a consequence, a partial convergence in performance was observed among the different formulations. Consistently, SEM–EDS observations performed on PB specimens revealed similar Ca content among all consolidants and less pronounced differences in consolidant distribution than those observed under HH conditions. Line scan profiles frequently showed preferential Ca localization within the outermost portion of the pictorial layer, suggesting that the higher saturation degree of the substrate limited consolidant migration towards deeper regions of the pore network. Despite these differences in distribution mechanisms, ΔDI values indicate that consolidating effectiveness remained significant for all products, with a slightly higher recovery for LAQ, although generally lower than under HH conditions. Moreover, the negative ΔDI values observed for control specimens treated with the aqueous dispersing media alone indicate that, under WC conditions, cohesion recovery cannot be attributed solely to the introduction of the liquid phase but is instead associated with the presence of the consolidating phase itself.

Similarly, all formulations reduced water uptake in the painted substrates despite the initially high saturation degree of the system. Aqueous dispersions exhibited the most pronounced reductions in ΔWa, particularly LAQ in PB specimens. The performance of LAQ highlights how aqueous dispersions may achieve effectiveness levels comparable to those of alcohol-based systems in terms of both cohesion recovery and reduction in water absorption. This behaviour may be associated with the different role assumed by the liquid phase under conditions close to capillary saturation. Under these conditions, the high water content within the porous network appears to limit the pathways available for consolidant transport toward deeper substrate regions. At the same time, the continuity between the pore water and the dispersing medium of aqueous consolidant may promote the mobility and redistribution of aqueous consolidants within the porous system.

The same dynamics associated with the presence of the liquid phase within the porous network also appear to be reflected in the chromatic behaviour of the investigated systems. From a colorimetric perspective, PS specimens exhibited limited variations for all formulations, with ΔE and ΔH values close to perceptibility thresholds, indicating substantial hue stability. In contrast, PB specimens showed marked chromatic variations for all treatments, including those performed using dispersing media alone. The high ΔE values, together with ΔH variations and the results obtained for dispersing media alone, suggest that chromatic variations were primarily governed by the presence of the liquid phase within the porous network rather than by the consolidant itself. Overall, the observed optical changes appeared to be mainly associated with generalized darkening phenomena rather than with superficial whitening effects. The presence of a liquid phase within the pore network may have modified the refractive index contrast between the solid matrix and pore space. This may have altered light-scattering mechanisms and the optical environment surrounding the pigment particles, in agreement with previous studies describing reflectance variations in wet multiple-scattering porous media [75].

## 5. Conclusions

This study provides a comparative assessment of different calcium-based consolidants applied to pictorial layers under environmental conditions representative of hypogean contexts. While the performance of calcium-based consolidants has been extensively investigated under dry or moderately humid conditions, their behaviour in environments approaching water saturation has received comparatively little attention. The findings contribute to addressing this gap by highlighting the critical influence of substrate moisture content on treatment performance.

The results demonstrate that consolidant effectiveness is governed by the complex interaction between the hygrometric state of the substrate, the nature of the dispersing medium, and the characteristics of the pictorial system. Both pre-treatment and efficacy tests suggest that maintaining continuity between the liquid phase within the substrate pore network and the dispersing medium of the consolidant may represent an important factor governing treatment performance. Under high-humidity conditions, alcohol-based formulations provided the greatest recovery of cohesion, whereas under water-content conditions the high degree of substrate saturation progressively reduced the differences among the tested formulations. In these circumstances, aqueous nanolime dispersions showed slightly more promising results.

The different responses observed under HH and WC conditions demonstrate that consolidant behaviour cannot be assessed independently from the hygrometric state of the porous system. In this respect, the study highlights the importance of adopting testing protocols and evaluation methods capable of reproducing realistic conservation conditions, thereby enabling a more reliable assessment of consolidant performance. These findings underline the need for application protocols specifically designed for highly humid conservation environments, such as hypogean settings, where substrate moisture plays a decisive role in determining treatment efficacy.

This study aims to provide conservators with a practical framework for informed selection of treatment strategies based not only on the intrinsic performance of a consolidant but also on the requirements of a specific conservation context, including environmental conditions, operational constraints and potential side effects that may influence treatment effectiveness.

## Figures and Tables

**Table 1:** Support mortar and paint layer compositions of specimen series (parts by volume, p.v.).

Specimen Series	Layer	Composition (p.v.)	Series ID
Painted–Siena earth	Support mortar	1 lime putty + 3 aggregate; fractions: 6 (250–500 μm); 3 (500–1000 μm); 1 (<250 μm)	PS
	Paint layer	1 Siena earth + 1.5 water	
Painted–ultramarine blue	Support mortar	1 lime putty + 3 aggregate; fractions: 6 (250–500 μm); 3 (500–1000 μm); 1 (<250 μm)	PB
	Paint layer	1 ultramarine blue + 1.5 water	

**Table 2:** Calcium-based products used in the study.

Product	Solid Phase	Dispersion Medium	Conc. (g/L) *	Particle Range Size *	Product ID
Nanorestore Plus^®^	Ca(OH)_2_	isopropanol	5	100–250 *	NNR
CaLoSiL^®^ IP	Ca(OH)_2_	isopropanol	5	80–150 *	CLS
Nanolaq^®^	Ca(OH)_2_	water	5	20–80 *	LAQ
Nanocalcite	CaCO_3_	water	5	15–40 **	NNC

* Particle size ranges reported according to Ref. [48]; ** particle size range reported according to data sheet.

**Table 3:** Testing moisture conditions and average specimen water-content values (mean ± SD).

Specimen Series	Conditions	T (°C), RH (%)	Water Content (Mean wt%)	Conditions ID
PS; PB	High-Humidity	14–15, 95–98	2.2 ± 0.78	HH
PS; PB	Water-Content	15, ~100	7.75 ± 1.44	WC

**Table 4:** Product application protocol under High-Humidity (HH) conditions.

	Consolidation Treatment				Pre-Treatment	Post-Treatment
Specimen Series	Total Volume (mL)	Number of Cycles	Product Concentration g/L (Cycles **)	Application Method * (Cycles **)	Solvent; Application Method * (Cycles **)	Solvent; Application Method * (Cycles **)
PS	52	8	5 g/L (1–8)	S (1–4) B + T (5–8)	IPA; S (1–4) IPA; B + T (5–8)	water; S (1–8)
PB	52	8	5 g/L (1–8)	S (1–4) B + T (5–8)	IPA; S (1–4) IPA; B + T (5–8)	water; S (1–8)

* S = spraying; B + T = brush + tissue paper. ** Numbers in parentheses indicate treatment cycle.

**Table 5:** Product application protocol under Water-Content (WC) conditions.

	Consolidation Treatment				Pre-Treatment	Post-Treatment
Specimen Series	Total Volume (mL)	Number of Cycles	Product Concentration g/L (Cycles **)	Application Method * (Cycles **)	Solvent; Application Method * (Cycles **)	Solvent; Application Method * (Cycles **)
PS	32	5	5 g/L (1–5)	S (1–2) B + T (3–5)	-	-
PB	32	5	5 g/L (1–5)	S (1–2) B + T (3–5)	-	-

* S = spraying; B = brush; B + T = brush + tissue paper. ** Numbers in parentheses indicate treatment cycles.

**Table 6:** Effect of surface pre-treatment conditions on white haze formation under HH and WC conditions after consolidation treatment. Values are expressed as %Area, corresponding to the percentage of threshold-selected pixels associated with white haze formation. Lower values indicate reduced whitening.

	HH Condition				WC Condition			
Pre-Treatment	NNR (%)	CLS (%)	LAQ (%)	NNC (%)	NNR (%)	CLS (%)	LAQ (%)	NNC (%)
No Pre-treatment	16.55	22.61	12.23	7.72	1.17	0.98	0.80	1.39
H_2_O	35.89	33.91	10.27	5.48	1.09	1.35	2.06	3.64
IPA	3.33	6.68	5.17	1.39	1.08	1.74	7.89	6.39
Acetone	8.00	6.83	8.24	10.77	4.56	5.65	10.93	10.15

**Table 7:** Colorimetric variations measured under HH conditions after treatment application. Values are reported as ΔE (means ± SD) and ΔH values.

	HH Condition			
Treatment	PS ΔE	PS ΔH	PB ΔE	PB ΔH
NNR	2.21 ± 0.46	0.77	8.71 ± 1.30	1.52
CLS	1.88 ± 1.09	0.45	7.80 ±1.37	1.24
LAQ	6.21 ± 0.66	0.68	12.42 ± 0.88	2.71
NNC	10.89 ± 0.84	2.14	16.56 ± 2.32	3.87
H_2_O	1.32 ± 0.43	0.45	0.72 ± 0.32	0.64
IPA	0.09 ± 0.26	0.09	2.45 ± 0.55	0.87
H_2_O + Biotin T	1.02 ± 0.18	0.50	0.87 ± 0.22	0.42

**Table 8:** Colorimetric variations measured under WC after treatment application. Values are reported as ΔE (means ± SD) and ΔH values.

	WC Condition			
Treatment	PS ΔE	PS ΔH	PB ΔE	PB ΔH
NNR	1.25 ± 0.63	0.13	6.47 ± 1.35	2.52
CLS	1.11 ± 0.72	0.19	6.78 ± 0.73	2.53
LAQ	1.25 ± 0.32	0.04	12.18 ± 0.69	3.80
NNC	1.47 ± 0.79	0.18	11.40 ± 1.06	2.99
H_2_O	1.05 ± 0.41	1.04	8.86 ± 0.67	7.69
IPA	0.53 ± 0.64	0.53	9.00 ± 0.38	7.14
H_2_O + Biotin T	0.80 ± 0.54	0.80	10.19 ± 0.36	7.91

**Table 9:** Relative variations in water absorption (Δwa) measured after treatments under HH and WC conditions. Values are expressed as percentage variations (mean ± SD) relative to untreated specimens.

	HH Condition		WC Condition	
Treatment	PS ΔWa (%)	PB ΔWa (%)	PS ΔWa (%)	PB ΔWa (%)
NNR	56 ± 17	33 ± 19	46 ± 14	54 ± 3
CLS	61 ± 9	35 ± 18	48 ± 7	56 ± 13
LAQ	57 ± 15	42 ± 10	59 ± 17	70 ± 9
NNC	29 ± 13	12 ± 29	60 ± 8	63 ± 6
**Control**				
H_2_O	22 ± 2	12 ± 4	22 ± 4	24 ± 8
IPA	14 ± 5	4 ± 10	14 ± 7	10 ± 18
H_2_O + Biotin T	18 ± 10	13 ± 17	21 ± 10	21 ± 8

**Table 10:** Relative water vapour diffusion resistance factor (Δμ) induced by the treatments under HH condition. Values are expressed as percentage variations (mean ± SD) relative to untreated specimens.

	HH Condition	
Treatment	PS Δμ (%)	PB Δμ (%)
NNR	−25 ± 20	−63 ± 26
CLS	−32 ± 31	−56 ± 27
LAQ	−21 ± 35	−21 ± 23
NNC	−41 ± 16	−52 ± 20
**Control**		
H_2_O	-	-
IPA	-	-
H_2_O + Biotin T	-	-

**Table 11:** Cohesion recovery after treatment under HH and WC conditions, expressed as percentage variations (mean ± SD) relative to untreated specimens (decohesion index = ΔDI%).

	HH Condition		WC Condition	
Treatment	PS ΔDI (%)	PB ΔDI (%)	PS ΔDI (%)	PB ΔDI (%)
NNR	72 ± 7	90 ± 9	55 ± 4	57 ± 4
CLS	93 ± 1	91 ± 4	51 ± 6	61 ± 6
LAQ	64 ± 16	78 ± 17	63 ± 6	72 ± 5
NNC	57 ± 11	76 ± 15	55 ± 5	48 ± 7
**Control**				
H_2_O	19 ± 14	44 ± 14	−12 ± 11	−12 ± 20
IPA	8 ± 18	27 ± 22	5 ± 15	13 ± 15
H_2_O + Biotin T	22 ± 37	45 ± 33	−6 ± 18	−8 ± 6

**Table 12:** Overall performance ranking of the tested consolidants under HH condition for PB and PS specimens, considering the main physicochemical and hygric properties investigated in this study.

HH Condition							
Products	Water Absorption Reduction		Colorimetric Stability		Cohesion Recovery		Ca Enrichment
	PS	PB	PS	PB	PS	PB	PB
NNR	+ +	-	+ + +	-	+ +	+ + +	+ +
CLS	+ + +	-	+ + +	+	+ + +	+ + +	+ + +
LAQ	+ +	+	+	- -	+ +	+ +	- -
NNC	-	- -	-	- - -	+	+ +	- - -

Rating symbols: - - - Very Poor, - - Poor, - Fair, + Good, + + Very Good, + + + Excellent.

**Table 13:** Overall performance ranking of the tested consolidants under WC condition for PB and PS specimens, considering the main physicochemical and hygric properties investigated in this study.

WC Condition							
Products	Water Absorption Reduction		Colorimetric Stability		Cohesion Recovery		Ca Enrichment
	PS	PB	PS	PB	PS	PB	PB
NNR	+	+ +	+ + +	+	+	+	- - -
CLS	+	+ +	+ + +	+	+	+	- - -
LAQ	+ + +	+ + +	+ + +	- -	+ +	+ +	- -
NNC	+ + +	+ + +	+ + +	- -	+	+	- -

Rating symbols: - - - Very Poor, - - Poor, - Fair, + Good, + + Very Good, + + + Excellent.

## Data Availability

The original contributions presented in this study are included in the article/Supplementary Materials. Further inquiries can be directed to the corresponding author.

## Supplementary Materials

The following supporting information can be downloaded at: https://www.mdpi.com/article/10.3390/nano16130831/s1, S1. Additional figures: Figure S1. XRD pattern of natural Siena earth pigment powder. Figure S2. XRD pattern of artificial ultramarine blue pigment powder; Figure S3. Mock-ups preparation: (a) square molds for plaster application, (b) plaster application, (c) mock-up plasters during drying, (d) application of paint layer by brush; S2. Additional tables: Table S1. Surface pH measurements of treated specimens under HH conditions. Table S2. Surface pH measurements of treated specimens under WC conditions. Table S3. Threshold intervals adopted for the qualitative ranking of consolidants performance. Table S4. Complete CIELAB parameter variations (ΔL*, Δa*, Δb*, ΔC*) under HH conditions. Table S5. Complete CIELAB parameter variations (ΔL*, Δa*, Δb*, ΔC*) under WC conditions.
