# Supplementary material for: Consolidation of Painted Plasters in Hypogean Environments: Comparative Performance of Inorganic Calcium-Based Products Under High-Humidity and Water-Saturated Conditions
Source: Nanomaterials (Basel). 2026 Jul 7;16(13):831. doi: 10.3390/nano16130831 (PMC13363546; doi:10.3390/nano16130831)
Supplement: Supplementary file 1 [file nanomaterials-16-00831-s001.zip › nanomaterials-4379160-supplementary.pdf]

Article

Consolidation of Painted Plasters in Hypogean Environments: Comparative Performance of Inorganic Calcium-Based Products Under High-Humidity and Water-Saturated Conditions

Roberta Cucchietti [1,2,†], Sara De Angelis [2,3,†], Eleonora Imperio [4], Vanessa Fontani [2,5], Lucia Conti [3], Giancarlo Sidoti [3] and Sara Iafrate [1,*]

[1] Wall Paintings Restoration Laboratory, Istituto Centrale per il Restauro (ICR), 00153, Rome, Italy; roberta.cucchietti@cultura.gov.it
[2] Department of Environmental Biology, Sapienza University of Rome, 00185, Rome, Italy; sara.deangelis-01@cultura.gov.it (S.D.A.); vanessa.fontani@cultura.gov.it (V.F.)
[3] Materials Testing Laboratory, Istituto Centrale per il Restauro (ICR), 00153, Rome, Italy; giancarlo.sidoti@cultura.gov.it (G.S.)
[4] Non-Destructive Testing Laboratory, Istituto Centrale per il Restauro (ICR), 00153, Rome, Italy; eleonoraimperio@yahoo.it
[5] Physics Laboratory, Istituto Centrale per il Restauro (ICR), 00153, Rome, Italy
* Correspondence: sara.iafrate@cultura.gov.it
† These authors contributed equally to this work.

Supplemetary Materials

This supplementary information includes additional data, tables and figures supporting the results presented in the main text.

S1. Additional Figure

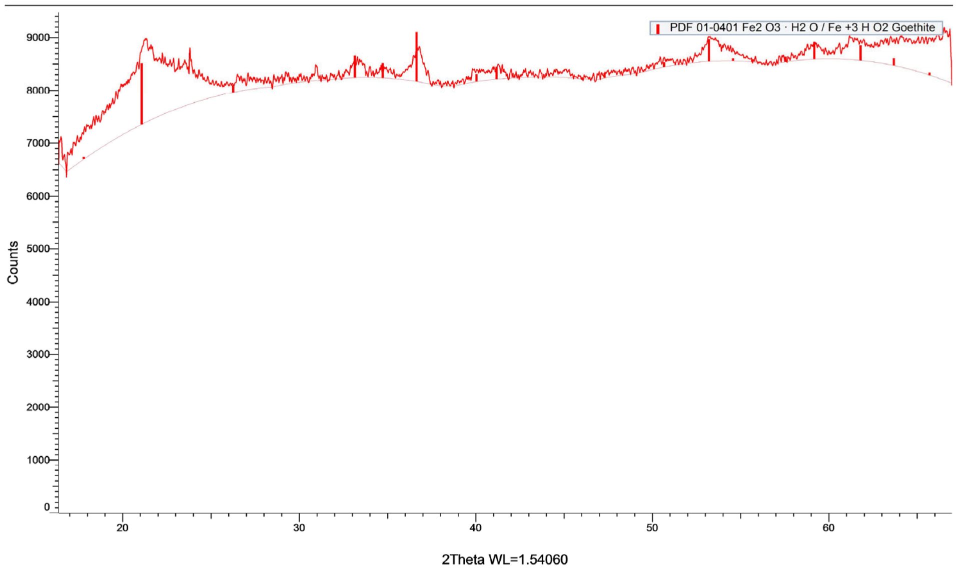

Figure S1. XRD pattern of natural Siena earth pigment powder.

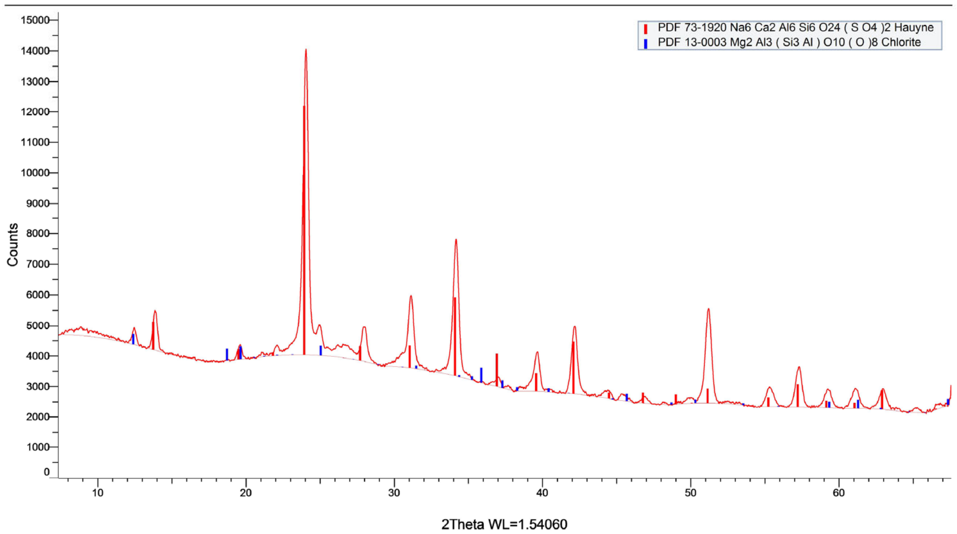

Figure S2. XRD pattern of artificial ultramarine blue pigment powder.

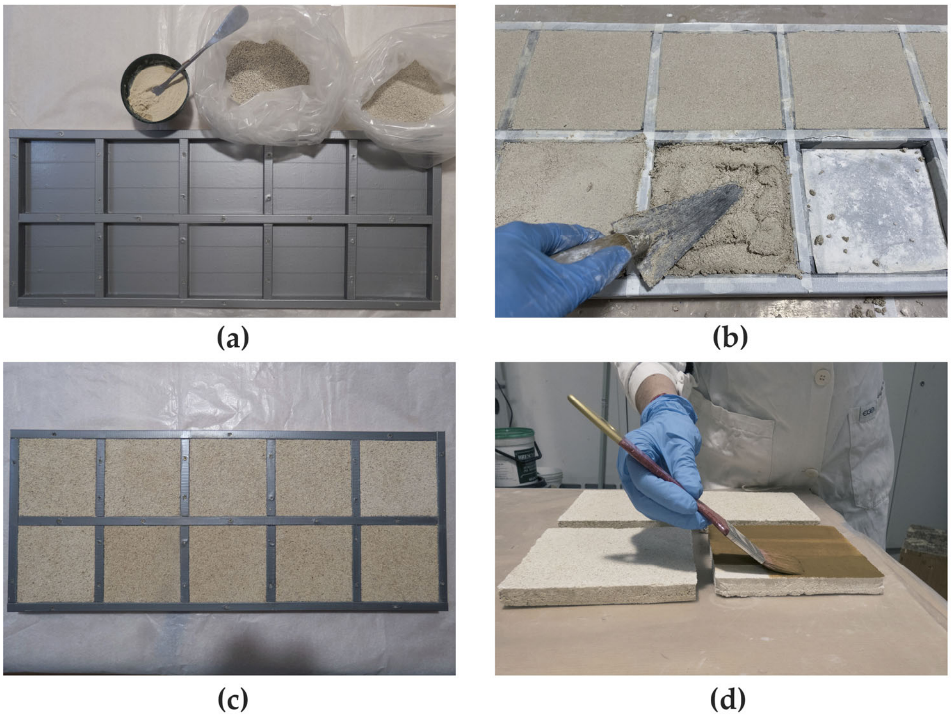

Figure S3. Mock-ups preparation: (a) square molds for plaster application, (b) plaster application, (c) mock-up plasters during drying, (d) application of paint layer by brush.

S2. Additional tables

Table S1. Surface pH measurements of treated specimens under HH conditions.

HH condition							
**Sample**	**Untreated**	**Immediately after treatment**			**30 days after treatment**		
		NNR	CLS	LAQ	NNR	CLS	LAQ
PS	8±0,1	12±0,2	12,2±0,4	11,6±0,4	8,2±0,4	8,1±0,2	8,2±0,2
PB	7,9±0,1	12,3±0,2	12,4±0,2	11,9±0,2	8,2±0,1	8,2±0,2	8,0±0,2

Table S2. Surface pH measurements of treated specimens under WC conditions.

WC condition							
**Sample**	**Untreated**	**Immediately after treatment**			**30 days after treatment**		
		NNR	CLS	LAQ	NNR	CLS	LAQ
PS	8±0,1	11,8±0,1	11,8±0,1	11,2±0,2	8±0,2	8,1±0,2	8,1±0,2
PB	8,3±0,2	11,6±0,2	11.6±0,2	11.6±0,2	8.1±0.2	8.2±0,2	8.2±0,2

Table S3. Threshold intervals adopted for the qualitative ranking of consolidants performance

**Parameter**	**Interval range**	**Rating**	**Meaning**
**Colorimetric stability (ΔE)***	0-2.8	+ + +	Excellent
	2.8-5.6	+ +	Very Good
	5.6-8.4	+	Good
	8.4-11.2	-	Fair
	11.2-14	- -	Poor
	14-16.8	- - -	Very Poor
**Cohesion recovery ΔDI (%)**	0-15.5	- - -	Very Poor
	15.5-31	- -	Poor
	31-46.5	-	Fair
	46.5-62	+	Good
	62-77.5	+ +	Very Good
	77.5-93	+++	Excellent
**Water absorption reduction Δwa (%)**	0-11.6	- - -	Very Poor
	11.6-23.3	- -	Poor
	23.3-35	-	Fair
	35-46.7	+	Good
	46.7-58.3	+ +	Very Good
	58.3-70	+ + +	Excellent
**Ca enrichment *Δ*Ca content(%)***	0-2.67	- - -	Very Poor
	2.67-5.34	- -	Poor
	5.34-8	-	Fair
	8-10.68	+	Good
	10.68-13.35	+ +	Very Good
	13.35-16	+ + +	Excellent

*ΔCa content(%): Ca content treated(%) - Ca content untreated(%)

Table S4. Complete CIELAB parameter variations (ΔL*, Δa*, Δb*, ΔC*) under HH conditions

	HH condition							
**Treatment**	**PS**				**PB**			
	**ΔL***	**Δa***	**Δb***	**ΔC***	**ΔL***	**Δa***	**Δb***	**ΔC***
NNR	-0.62	-0.16	-2.12	-1.98	1.94	-4.11	7.43	-8.36
CLS	0.05	-0.38	-1.84	-1.83	2.07	-3.59	6.60	-7.41
LAQ	2.27	-1.86	-5.47	-5.74	5.47	-5.96	9.43	-10.82
NNC	3.03	-2.86	-10.11	-10.24	7.74	-7.95	12.29	-14.12
**Control**								
H_2O	-0.42	0.41	1.18	1.24	-0.61	-0.29	0.26	-0.34
IPA	0.06	-0.04	0.06	0.04	0.48	-1.41	1.95	-2.30
H_2O+Biotin	-0.50	0.33	0.82	0.88	-0.10	-0.62	0.60	-0.76

Corresponding ΔE* and ΔH* values are reported in Tables 8 of the main text

Table S5. Complete CIELAB parameter variations (ΔL*, Δa*, Δb*, ΔC*) under WC conditions

	WC condition							
**Treatment**	**PS**				**PB**			
	**ΔL***	**Δa***	**Δb***	**ΔC***	**ΔL***	**Δa***	**Δb***	**ΔC***
NNR	-1.23	-0.20	-0.09	-0.18	-4.74	0.95	4.30	-3.61
CLS	-1.09	-0.20	0.04	-0.07	-5.54	1.22	3.70	-2.96
LAQ	-0.99	-0.42	0.63	-0.76	-7.93	0.21	9.24	-8.43
NNC	-0.82	-0.46	1.13	-1.21	-6.76	-0.57	9.16	-8.68
**Control**								
H_2O	-1.04	0.14	0.06	0.12	-6.52	2.01	5.66	-4.41
IPA	-0.48	0.24	-0.04	0.09	-6.09	1.26	6.51	-5.49
H_2O+Biotin	-0.79	0.12	-0.03	0.04	-6.79	1.21	7.50	-6.42

Corresponding ΔE* and ΔH* values are reported in Tables 9 of the main text
